# Mesenchymal stem cell exosomes reverse acute lung injury through Nrf-2/ARE and NF-*κ*B signaling pathways

**DOI:** 10.7717/peerj.9928

**Published:** 2020-09-18

**Authors:** Jun Li, Xingqi Deng, Xiangling Ji, Xiaojun Shi, Zhiying Ying, Kan Shen, Dongwei Xu, Zhihui Cheng

**Affiliations:** 1 South Hospital of the Sixth People’s Hospital Affiliated to Shanghai Jiaotong University, Shanghai, China; 2 Shanghai University of Medicine & Health Sciences Affiliated Zhoupu Hospital, Shanghai, China

**Keywords:** Acute lung injury, Mesenchymal stem cell, Exosome

## Abstract

Acute lung injury (ALI) is associated with histopathological diffuse alveolar damage. The potential role of mesenchymal stem cells (MSCs) in the treatment of various clinical disorders have been widely documented, such as those for ALI. Recent evidence has demonstrated that exosomes from endothelial progenitor cells can improve outcomes of the lipopolysaccharide (LPS)-induced ALI. However, there has been no research on the potential role of MSC-exosomes in the treatment of sepsis-induced ALI, which is worth further exploration. Thus, the objective of our study was to identify whether the MSC-exosomes could reverse ALI. The ALI model induced by LPS was established in this study. MTT assay was performed to test cell proliferation. Expression of inflammatory factors (TNF-α, IL-6, and IL-10) in the LPS-treated type II alveolar epithelial cells (AECs) (MLE-12) was detected by ELISA. After co-culture of MSC-exosomes with LPS-treated MLE-12 cells, we found that the cell proliferation of MLE-12 cells gradually increased. Furthermore, we selected five of the Nrf-2/ARE- and NF-κB signaling pathway-related genes to explore if MSC-exosomes could reverse LPS-induced ALI through Nrf-2/ARE and NF-κB signaling pathways. QRT-PCR and western blot experiment results showed that the expression of these five genes were significantly regulated after stimulation with high-concentration LPS and exosome intervention. Taken together, these findings highlighted the fact that MSC-exosomes could reverse ALI through the Nrf-2/ARE and NF-κB signaling pathways. The MSC-exosome may be the potential future therapeutic strategy for the treatment of ALI.

## Introduction

Sepsis is a disease common worldwide with high mortality rates, which usually results from the dysregulated host response to infection (Rhodes, Evans & Alhazzani, 2017). It has been reported that approximately 40% of the acute lung injury (ALI) incidences are caused by sepsis (Stapleton et al., 2005). ALI is associated with histopathological diffuse alveolar damage. At present, there is no effective treatment strategy for sepsis-induced ALI. Therefore, we should explore new treatment strategies for sepsis-induced ALI.

Mesenchymal stem cell (MSC) are stem cells which always reside in adult tissues and have the capability to undergo unlimited amplification and multipotent differentiation (Ding, Shyu & Lin, 2011). It has been reported that MSCs can modulate the host immune response to injury and infection and promote repair following tissue injury (Liu, Pan & Liang, 2014). Previous research results indicated that the therapeutic role of MSCs was mediated by exosomes (Rager, Olson & Zhou, 2016). Recent research has shown that MSC-exosomes can play effective roles in various tissue injuries (Phinney & Pittenger, 2017). However, there are no reports about the potential roles of MSC-exosomes in sepsis-induced ALI.

Exosomes are nano-sized vesicles (40–100 nm) released from a variety of cells (Lasser, 2012). Recently, it has been reported that exosomes can play important roles in the treatment of lung cancer or various lung injuries (Masaoutis et al., 2018; Kojima et al., 2018). Some evidences have indicated that MSC-derived exosomes (MSC-exosomes) can play roles in the treatment of chronic inflammation and contribute to the enhancement of the therapeutic effect of MSCs (Ti et al., 2016). Recently a study indicated that exosomes from endothelial progenitor cells can improve outcomes of the LPS-induced ALI (Zhou et al., 2019). However, there is no evidence about the potential roles of MSC-exosomes in the treatment of sepsis-induced ALI, which is worth further exploration. Thus, the objective of our study was to identify whether the MSC-exosomes could reverse sepsis-induced ALI. Moreover, we also obtained data verifying whether MSC-exosomes could reverse sepsis-induced ALI through Nrf-2/ARE and NF-κB signaling pathways. This study provided the theoretical basis for the targeted treatment of sepsis-induced ALI.

## Materials & Methods

### In vitro MSC isolation

Female SPF grade C57BL/6 mice (weight 24–26 g) were obtained from Sino-British SIPPR/BK Lab Animal Ltd. (Shanghai, China). Mice were fed in IVC respiratory system and provided with drinking water. Room temperature was maintained at 25 °C with 12-h light/12-h dark cycle. Mice were anesthetized prior to killing by cervical dislocation and were exposed to isoflurane in the airflow chamber with 500 ml/min airflow and 3% isoflurane. The animal procedures were conducted in accordance with the NIH guide of Humane Use and Care Animals, and were approved by The Animal Care and Use Committee of the Shanghai University of Medicine & Health Sciences (240273). After sacrifice, marrow cells were obtained from C57BL/6 mice, and then centrifuged at 1500 rpm for 5 minutes. Supernatant was discarded and the precipitate was resuspended in DMEM culture medium containing 10% FBS. The isolated MSCs were cultured in an incubator at 37 °C containing 5% CO2.

### Isolation and identification of MSC-exosomes

The MSCs were cultured in DMEM. Exosomes were obtained according to the manufacturer’s instructions (exoEasy Maxi Kit, QIAGEN). The MSCs were subjected to TEM analysis as follows: the exosomes in PBS were dropped on gilder grids with continuous carbon film above for about 20 mins, and then were stained with uranyl acetate and lead citrate. Finally, the stained exosomes were examined with the Hitachi-800 transmission electron microscope (Hitachi, Japan).

### LPS induction in alveolar epithelial cells (AECS)

MLE-12 cell line (The type II AECs) was purchased from the Chinese Academy of Sciences (Shanghai, China). MLE-12 cells were treated with LPS (Sigma, St. Louis, MO, USA). Then, the cells were cultured at 37 °C in an incubator containing 5% CO_2_.

### Quantitative real-time polymerase chain reaction (QRT-PCR) assay

Total RNA was extracted from cells using the TRIzol kit (Invitrogen). One μ g of the total RNA was reverse transcribed into cDNA. QRT-PCR of the samples was performed using the SYBR^®^ Premix Ex Taq™ kit (Takara). The primers used are shown in Table 1. Beta-actin was used as the internal control.

**Table 1:** Primers and antibodies.

Company	Item number	Name	Primer	Sequence (5′–3′)
abcam	ab62352	Anti-Nrf2 [EP1808Y]	GR-F	CTACCCTGGTGTCACTGCTG
abcam	ab13248	Anti-Heme Oxygenase 1[HO-1-1]	GR-R	TGGTATCGCCTTTGCCCATT
abcam	ab216130	Anti-CD63	HO-1-F	ATGCCCCACTCTACTTCCCT
cst	10037S	CD81	HO-1-R	TTTGAACTTGGTGGGGCTGT
abcam	ab22604	Anti-Glutathione Peroxidase 1	GPX-1-F	GGACACCAGGAGAATGGCAA
abcam	ab2768	Anti-Glucocorticoid Receptor [BuGR2] - ChIP Grade	GPX-1-R	AAGGTAAAGAGCGGGTGAGC
abcam	ab86299	Anti-NF-kB p65 (phospho S536)	Nrf-2-F	GAGCAGGACATGGAGCAAGT
abcam	ab58989	CD9	Nrf-2-R	AGTGACTGACTGATGGCAGC
abcam	ab216647	CD44	NF-kB-F	ATGGCAGACGATGATCCCTAC
abcam	ab214437	CD45	NF-kB-R	TGTTGACAGTGGTATTTCTGGTG
abcam	ab54217	CD73	β-actin_F	ATCATGTTTGAGACCTTCAACA
abcam	ab8227	Anti-beta Actin	β-actin_R	CATCTCTTGCTCGAAGTCCA

### Flow cytometry

MSCs were resuspended at a density of 2 × 10^6^ cells/mL in staining buffer. All cells were stained with antibodies of extracellular markers (CD44, CD45, and CD73). The samples were run on the BD Accuri™ C6 (BD Bioscience) flow cytometer. The antibodies used are shown in Table 1.

### Western blot analysis

The cells were lysed in the RIPA buffer at 4 ^∘^C for 16 min and then centrifuged at 15000 rpm for 20 min. BCA assay was used for the protein concentration detection. Samples were separated by PAGE and transferred onto a PVDF membrane. The antibodies used are shown in Table 1. Beta-actin was used as an internal control.

### MTT assay

MTT dye reduction assay (Sigma, St.Louis, Mo, USA) was carried out to detect the cell viability as previously reported (Dai & etal, 2012). Briefly, cells were seeded into a 96-well plate at a density of 1 × 10^5^ cells/well, cultured for 12 h, then treated with LPS at a concentration of 300 μ g/mL for 24 h, and then treated with MSC-exosomes for 24 h. At the end of the treatment, 10 μ L (50 μ g) of the MTT solution was added into the cells and incubated for another 4 h. Two hundred microliters of dimethylsufloxide (DMSO), was added to each well after removal of the supernatant. After shaking for 10 min, cell viability was measured at an absorbance wavelength of 490 nm using an Enzyme-labeling instrument (Multiskan. Go, Thermo Scientific, Ratastie, Finland).

### ELISA detection

The supernatant levels of IL-10, IL-6, and TNF-α were quantified with the ELISA kit for each cytokine (Excellbio) according to the manufacturer’s instructions. First, 50 μ L of the diluted standard samples or supernatant samples were added into the microplate and were incubated at 37 °C for 1 h. Second, 4 ml antibody solution was added to each well against, TNF-α, IL-6, or IL-10 and incubated at 37 °C for another 1 h. Third, the plate was added with horseradish peroxidase-linked secondary antibody for incubation at 37 °C for 30 min. After washing four times with TBST, the plate was inoculated with substrate and was assayed immediately at 450 nm with a spectrophotometer (Bio-Rad Laboratories).

### Statistical analysis

Data were analyzed using the SPSS 19.0 software. Comparisons among multiple groups were analyzed by one-way analysis of variance (ANOVA). Comparisons of mean values between two groups were analyzed using a non-paired *t*-test.

## Results

### Isolation of MSCs and identification of its exosome

**Figure 1:**
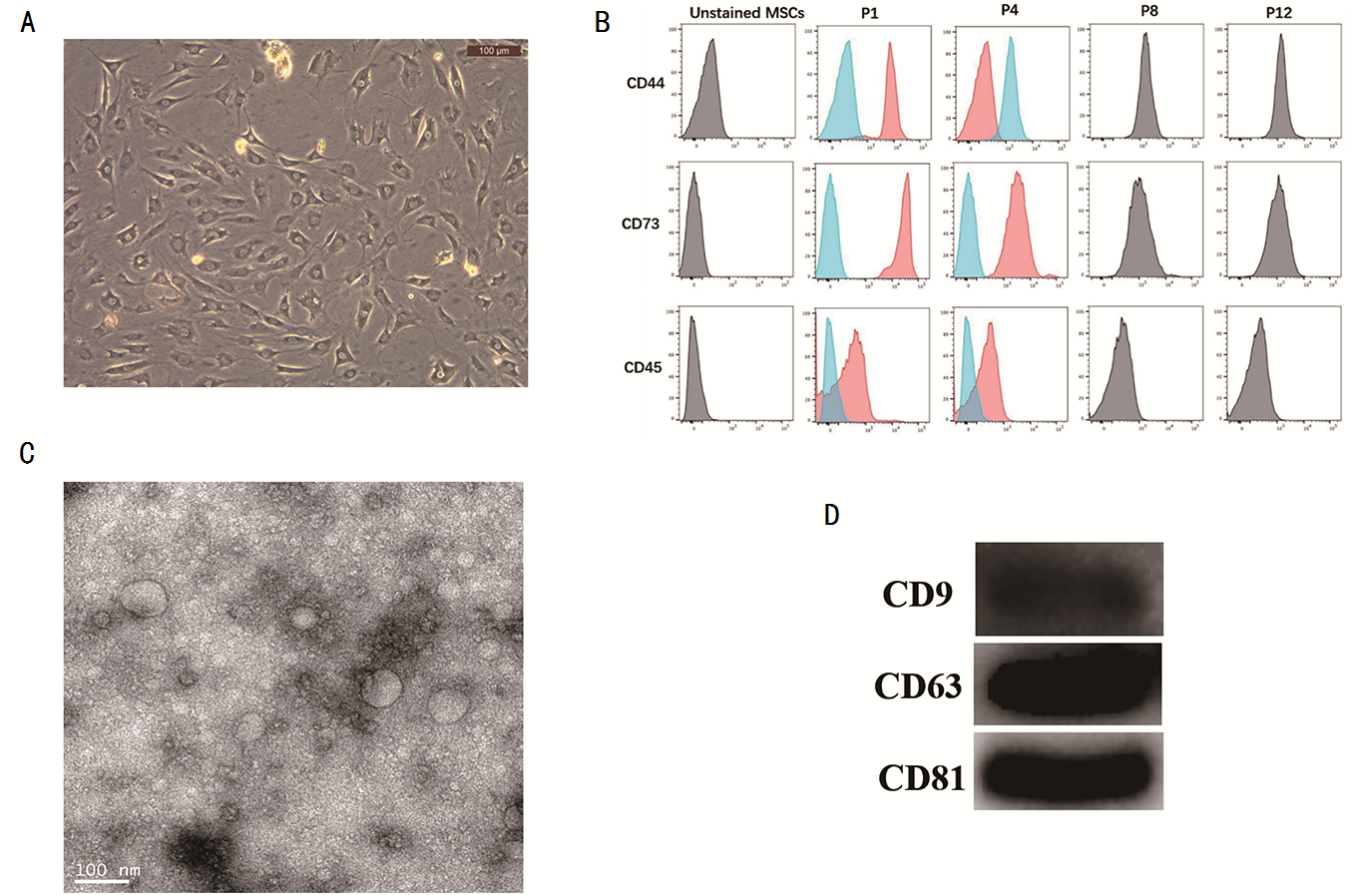
Isolation of MSCs and identification of its exosome. (A) Micrograph of the morphology of MSCs (B) Flow cytometry was used to detect the expression of CD44, CD73 and CD45. (C) TEM was used to observe the change in morphology of MSC-exosomes (20000X). (D) Western blot experiment was used to analyze the expression of CD9, CD63, and CD81 in MSC-exosomes. MSCs, mesenchymal stem cells. TEM, transmission electron microscopy.

MSCs were isolated and characterized. The cell morphology of MSC was observed under a microscope (Fig. 1A). The surface markers of MSCs like CD44, CD73, and CD45 were detected by flow cytometry (Fig. 1B), thus confirming that the MSCs had been successfully isolated. The MSC-exosomes were obtained and observed under a TEM microscope. They were observed to have round or oval membranous vesicles with a size ranging from 30 nm to 120 nm (Fig. 1C). Next, western blot experiment was performed to analyze the expression of CD9, CD63, and CD81 from MSC-exosomes (Fig. 1D).

**Figure 2:**
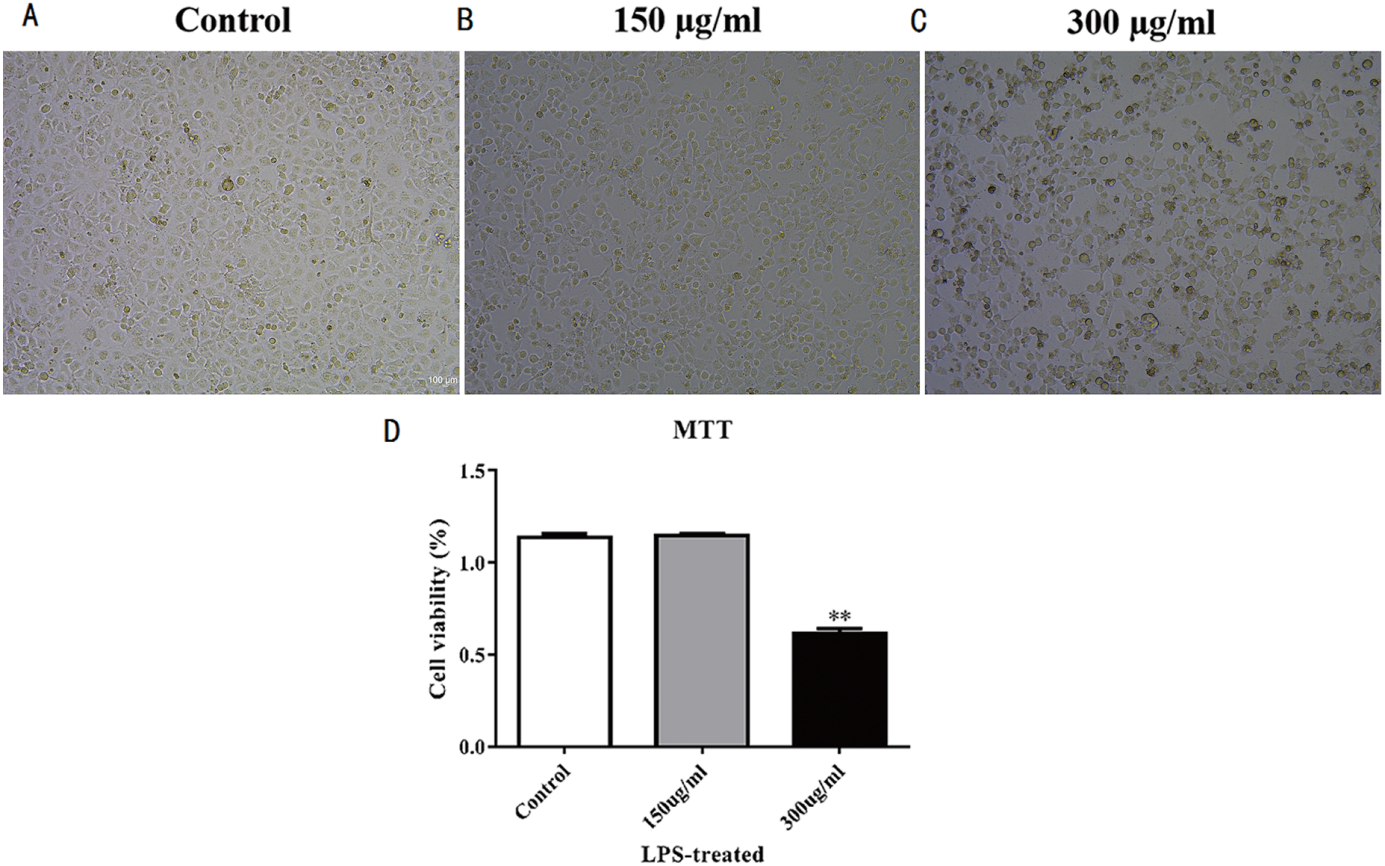
Establishment of LPS-induced acute lung injury model. (A–C) Cell proliferation morphology of each group after high-concentration LPS treatment. Three groups that were studied are as follows: control, 150 μ g/mL LPS-treated, and 300 μ g/mL LPS-treated. (D) MTT detection of MLE-12 cell proliferation after LPS treatment for 48 hours. The results showed the proliferation of MLE-12 cells in the 300 μ g/mL LPS-treated group slowed down.

### Establishment of the LPS-induced ALI model

MLE-12 cells were stimulated by LPS to establish the acute lung injury model. Results of concentration gradient test showed MLE-12 cells were most sensitive to 300 μ g/mL LPS; thus, we selected this LPS concentration to stimulate MLE-12 cells for acute lung injury model establishment. The cell morphology of LPS-treated MLE-12 cells were observed under the microscope (Figs. 2A–2C), and results showed the cell morphology of MLE-12 cells had changed. MTT detection was performed after LPS treatment for 48 h to test cell proliferation. Results showed that the proliferation of MLE-12 cells in the 300 µg/mL LPS-treated group slowed down markedly (Fig. 2D).

### MSC-exosomes could reverse LPS-induced ALI

**Figure 3:**
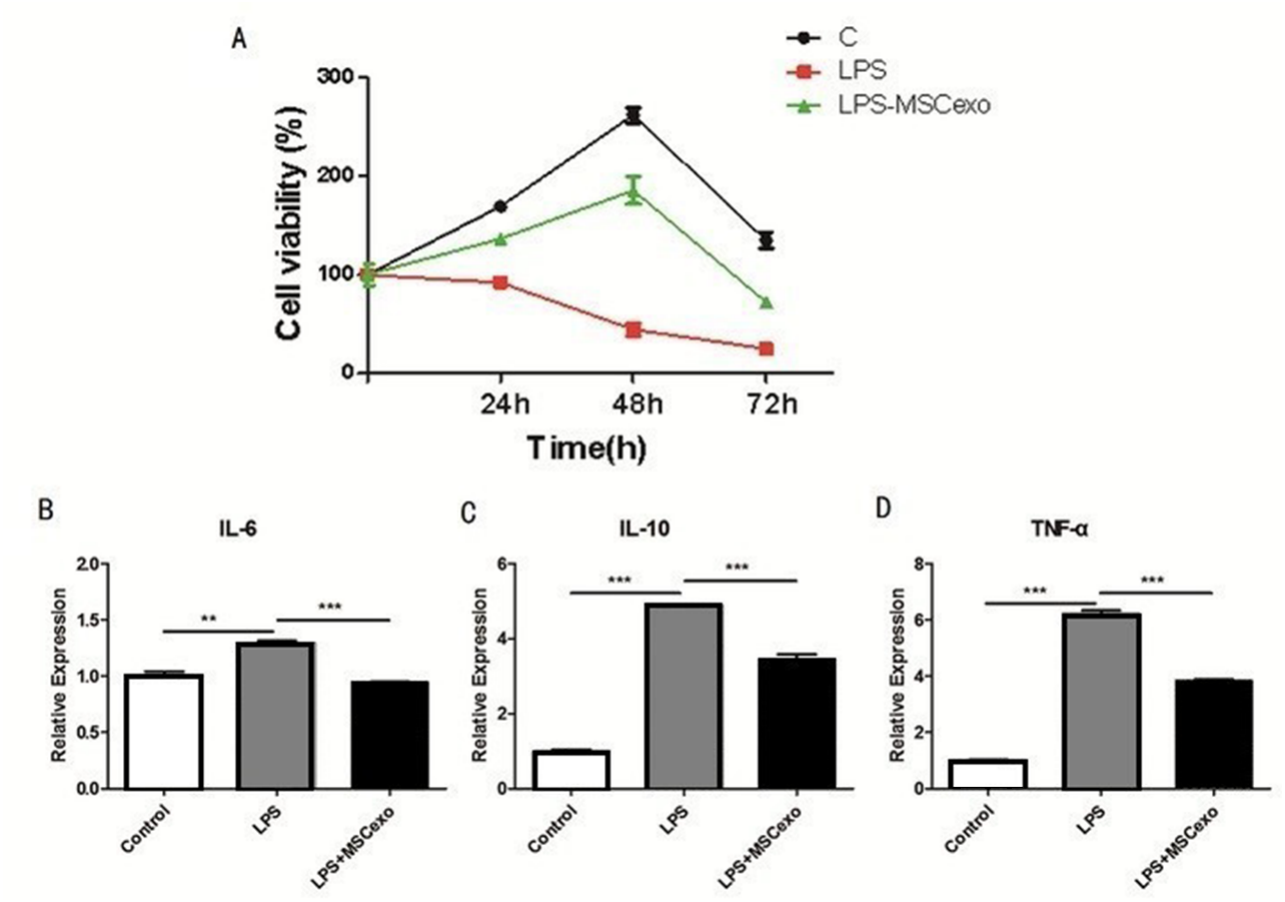
MSC-exosomes could reverse LPS-induced lung injury. (A) MLE-12 cells were treated with LPS and MSC-exosomes and detected by MTT to evaluate cell proliferation. The results showed the LPS-treated cells proliferated the slowest of all cells. (B–D). Expression of inflammatory factors (TNF-*α*, IL-6, and IL-10) in MLE-12 cells were detected by ELISA after high concentration LPS stimulation and exosomes intervention.

MLE-12 cells were treated with LPS and MSC-exosomes and detected by MTT to evaluate cell proliferation. The results showed that the LPS-MSC-exosome-treated cells proliferated slowly as compared to the control (Fig. 3A). Expression of inflammatory factors (TNF-α, IL-6, and IL-10) in MLE-12 cells was detected by ELISA, after high-concentration LPS stimulation and exosome intervention. The results showed that the expression of inflammatory factors in LPS-MSC-exosome-treated cells was significantly down-regulated as compared to the control LPS-treated group (Figs. 3B–3D).

### MSC-exosomes could reverse LPS-induced ALI through the Nrf-2/ARE and NF-κB signaling pathways

**Figure 4:**
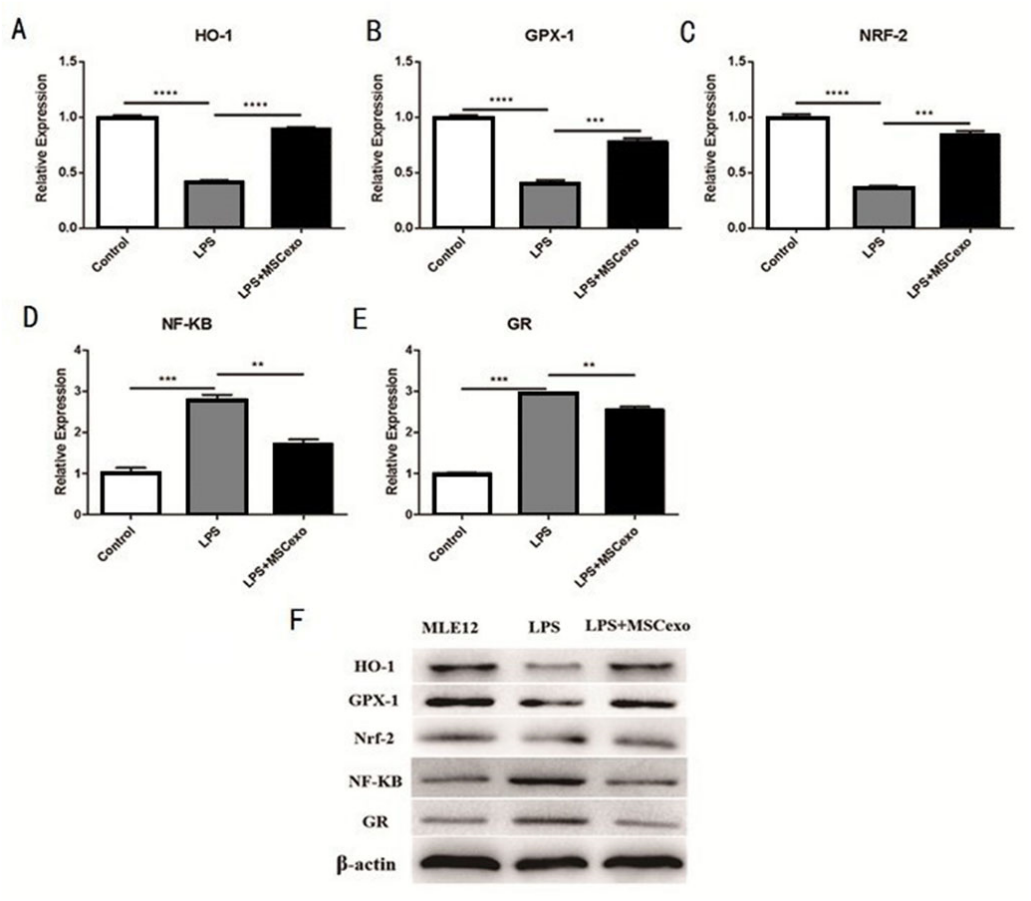
MSC-exosomes could reverse LPS-induced lung injury through Nrf-2 / ARE and NF-kB signaling pathways. (A–E) QRT-PCR results showed mRNA expression of these five genes (Ho-1, GPX-1, NRF-2, NF-kB, and GR) were all significantly regulated after high-concentration LPS stimulation and exosome intervention. (F) Western blot experiment was performed to analyze the protein expression of these five genes. Results showed that the expression of these five genes was significantly regulated after high-concentration LPS stimulation and exosome intervention.

We selected five Nrf-2/ARE- and NF-κB signaling pathway-related genes, including hmox1 heme oxygenase 1 (Ho-1), glutathione peroxidase 1 (GPX-1), nuclear factor, erythroid derived 2, like 2 (NRF-2), unclear factor kappa B subunit 1 (NF-kB), and glucocorticoid receptor (GR), to explore if MSC-exosomes could reverse LPS-induced acute lung injury through the Nrf-2/ARE and NF-κB signaling pathways. QRT-PCR results showed mRNA expression of these five genes were all significantly regulated after high-concentration LPS stimulation and exosomes intervention (Figs. 4A– 4E).Western blot experiment was performed to analyze the protein expression of these five genes, and results showed that expression of these five genes were also significantly regulated after high-concentration LPS stimulation and exosome intervention (Fig. 4F).

## Discussion

The ALI model induced by LPS was established in this study. The MSCs were identified by flow cytometry and their cell morphology was observed under the microscope. MTT assay was used to evaluate cell proliferation of MLE-12 cells after their treatment with LPS and MSC-exosomes. ELISA was used to detect expression of inflammatory factors (TNF-α, IL-6, and IL-10) in MLE-12 cells, after stimulation with high concentration LPS and exosomes intervention. The results indicated that MSC-exosomes could reverse LPS-induced ALI. Furthermore, we selected five Nrf-2/ARE- and NF-κB signaling pathway-related genes, including Ho-1, GPX-1, NRF-2, NF-κB and GR, to explore whether MSC-exosomes could reverse LPS-induced ALI through the Nrf-2/ARE and NF-κB signaling pathways. QRT-PCR and western blot experiment results showed that the expression of these five genes was significantly regulated after high-concentration LPS stimulation and exosome intervention. In conclusion, our data demonstrated that MSC-exosomes could reverse LPS-induced ALI through the Nrf-2/ARE and NF-κB signaling pathways.

Recent evidence suggested that MSC-exosomes could protect against ALI by activating a series of responses (Tan et al., 2014). However, there are few studies about the intercellular communication between MSCs and AECs, especially explaining the role of MSC-exosomes involved in the development of ALI. Hence, further studies should aim to demonstrate if MSCs can influence AECs via some RNAs or proteins enclosed by MSC-exosomes. Furthermore, the effect of MSC-exosomes for ALI should be further investigated.

## Conclusions

Our data demonstrated that MSC-exosomes could reverse LPS-induced ALI. Moreover, we found MSC-exosomes could reverse the process through the Nrf-2/ARE and NF-κB signaling pathways.

## Additional Information and Declarations

### Data Availability

The following information was supplied regarding data availability: The data are available at Figshare: DENG (2020): Raw data. figshare. Dataset. DOI: 10.6084/m9.figshare.11392911.v1.
